# Effect of Enamel Caries Lesion Baseline Severity on Fluoride Dose-Response

**DOI:** 10.1155/2017/4321925

**Published:** 2017-03-27

**Authors:** Frank Lippert

**Affiliations:** Indiana University School of Dentistry, Department of Cariology, Operative Dentistry and Dental Public Health, Oral Health Research Institute, Indianapolis, IN, USA

## Abstract

This study aimed to investigate the effect of enamel caries lesion baseline severity on fluoride dose-response under pH cycling conditions. Early caries lesions were created in human enamel specimens at four different severities (8, 16, 24, and 36 h). Lesions were allocated to treatment groups (0, 83, and 367 ppm fluoride as sodium fluoride) based on Vickers surface microhardness (VHN) and pH cycled for 5 d. The cycling model comprised 3 × 1 min fluoride treatments sandwiched between 2 × 60 min demineralization challenges with specimens stored in artificial saliva in between. VHN was measured again and changes versus lesion baseline were calculated (ΔVHN). Data were analyzed using two-way ANOVA (*p* < 0.05). Increased demineralization times led to increased surface softening. The lesion severity×fluoride concentration interaction was significant (*p* < 0.001). Fluoride dose-response was observed in all groups. Lesions initially demineralized for 16 and 8 h showed similar overall rehardening (ΔVHN) and more than 24 and 36 h lesions, which were similar. The 8 h lesions showed the greatest fluoride response differential (367 versus 0 ppm F) which diminished with increasing lesion baseline severity. The extent of rehardening as a result of the 0 ppm F treatment increased with increasing lesion baseline severity, whereas it decreased for the fluoride treatments. In conclusion, lesion baseline severity impacts the extent of the fluoride dose-response.

## 1. Introduction

The efficacy of topical fluorides in caries prevention has been demonstrated in many clinical trials and for a range of compounds and delivery formats [1]. However, fluoride's efficacy depends on when it is introduced in the caries process, as a conventional fluoride toothpaste was shown to be effective in preventing the onset of caries, but its effect was diminished in preventing the progression of radiographically detectable lesions [2]. Several entirely mechanistic studies [3, 4] have since shown this effect to be also true for shallower lesions than those often encountered clinically and pointed out that considerably higher fluoride concentrations are needed to prevent progression of lesions than their initiation. Paradoxically, both in situ [5–7] and in vitro caries studies [8] have been able to demonstrate “lesion baseline effects”; that is, initially less demineralized lesions are more prone for further progression than more demineralized ones, and vice versa; initially more demineralized lesions have greater capacity for remineralization than less demineralized ones. None of these studies, however, considered the comparison between placebo and fluoride, let alone fluoride dose-response effects.

A sole pH cycling study [9] could be retrieved that investigated lesion baseline effects on fluoride dose-response. An approximate 3.5-fold difference in baseline mineral loss had a somewhat muted effect on the ability of fluoride at various concentrations to induce remineralization and prevent further demineralization as a slightly better response to fluoride was shown for initially more demineralized lesions. The tested lesions, however, were already established lesions with a defined surface zone. During lesion formation intraorally, lesions are initially surface-softened and a mineralized surface zone typically only forms after several weeks and is subject to compositional and structural changes throughout the entire caries process [10]. Consequently, further research is warranted on the efficacy of fluoride at various concentrations on less developed, early caries lesions to better understand the relative efficacy of fluoride in preventing lesion progression at their earliest stage. Therefore, the aim of the present in vitro study was to investigate the effect of lesion baseline severity on fluoride dose-response in very early caries lesions under pH cycling conditions.

## 2. Materials and Methods

### 2.1. Study Design

The present laboratory study followed a 4 (lesion severity) × 3 (fluoride concentrations) factorial design, thus resulting in a total of 12 experimental groups. Early caries lesions were prepared in human enamel specimens employing demineralization times of 8, 16, 24, and 36 h. Lesions were assigned to treatment groups (0, 83, and 367 ppm fluoride) based on Vickers surface microhardness (VHN) and pH cycled for 5 d. VHN was determined again and the extent of rehardening or further surface softening calculated.

### 2.2. Specimen Preparation

Human tooth crowns were cut into 4 × 4 mm specimens using a Buehler Isomet low-speed saw with one specimen prepared per tooth. Human teeth were extracted mainly for orthodontic reasons and were obtained from dental offices located in the State of Indiana, USA (water fluoridation at approx. 1 ppm F). IRB approval was obtained prior to tooth collection (NS0911-07). Specimens were ground and polished to create flat, planar parallel dentin and enamel surfaces using a Struers Rotopol 31/Rotoforce 4 polishing unit (Struers Inc., Cleveland, Pa., USA). The dentin side of the specimens was ground flat to a uniform thickness with 500-grit silicon carbide grinding paper. The enamel side of the specimen was serially ground using 1,200-, 2,400-, and 4,000-grit paper. The specimens were then polished using a 1 *μ*m diamond polishing suspension on a polishing cloth until the enamel surface had a minimum of a 3 × 3 mm highly polished facet in the center of the specimen. This polishing procedure ensured the removal of surface enamel (amount depending on the natural curvature of the enamel surface) which may contain relatively high concentrations of artificially introduced trace elements (e.g., fluoride, strontium) that would otherwise compromise the study aims. The specimens were assessed under a Nikon SMZ 1500 stereomicroscope at 20x magnification for cracks, hypomineralized (white spots) areas, or other flaws in the enamel surface that would exclude them from use in the study. Specimens were stored at 100% relative humidity at 4°C throughout the study unless they were pH cycled or hardness measurements were performed. A total of 186 specimens were prepared.

### 2.3. Sound Enamel Surface Microhardness

Four sound enamel baseline indentations (2100 HT; Wilson Instruments, Norwood, Mass., USA) were placed in the center of each specimen using a Vickers diamond indenter using a 200 g load (approx. 150 *μ*m apart from each other), each with a dwelling time of 11 s. Vickers hardness numbers (VHN_sound_) were derived from the respective indentation lengths and recorded. Only specimens which fulfilled the criteria of 310 ≤ VHN_sound_ ≤ 380 (180 specimens) were included in the study and divided into four subgroups (*n* = 45 per group, one for each demineralization time) to ensure no significant differences in VHN_sound_ between subgroups.

### 2.4. Artificial Caries Lesion Creation

Artificial caries lesions were formed in the enamel specimens by an 8, 16, 24, or 36 h immersion into a solution of 0.1 M lactic acid, 0.2% Carbopol 907, 3.0 mM CaCl_2_  × 2H_2_O, 6.0 mM KH_2_PO_4_, 63.0 mM KCl, and 3.1 mM NaN_3_, pH adjusted to 5.0 using KOH.

### 2.5. Lesion Surface Microhardness and Treatment Group Balancing

The lesion surface microhardness was determined as described above by placing four indentations to the right of the sound enamel indentations (approx. 150 *μ*m apart from each other), yielding VHN_lesion_. From each subgroup of 45 specimens, 36 were selected and divided further into three treatment groups (0, 87, and 383 ppm fluoride) of 12 specimens each. The initial selection of 36 specimens from each subgroup was based on selecting specimens whose VHN_lesion_ was closest to the mean VHN_lesion_ within each subgroup. Further division into treatment groups was performed to ensure no significant differences in VHN_lesion_ between treatment groups within each subgroup.

### 2.6. pH Cycling Phase

The pH cycling regimen is presented in Table 1. Lesions were pH cycled for a total of 5 d. The daily pH cycling regimen included three 1 min interventions (treatments with the test solutions), sandwiched around two blocks of 60 min remineralization (artificial saliva), 60 min demineralization, and 60 min remineralization with overnight remineralization after the last intervention. Treatment solutions were aqueous sodium fluoride solutions differing only in fluoride concentration, 0, 87, and 383 ppm fluoride, thereby mimicking placebo, 250 ppm and 1150 ppm fluoride toothpastes after 1 : 3 dilution. Solutions had a pH value of 5.5–6.0. The composition of artificial saliva was 1.5 mM CaCl_2_  × 2H_2_O; 0.9 mM KH_2_PO_4_; 130.0 mM KCl; 20.0 mM HEPES; 3.1 mM NaN_3_, adjusted to pH 7.0 with KOH, whereas the demineralization solution had the following composition: 50.0 mM acetic acid; 2.2 mM CaCl_2_  × 2H_2_O; 2.2 mM KH_2_PO_4_; 3.1 mM NaN_3_, adjusted to pH 5.0 with KOH. Lesions were rinsed with deionized water after each treatment or solution change.

### 2.7. Post-pH Cycling Surface Microhardness

The post-pH cycling lesion surface microhardness was determined as described above by placing four indentations to the left of the sound enamel indentations (approx. 150 *μ*m apart from each other), yielding VHN_post_. Changes in VHN were calculated for each specimen as follows: ΔVHN = VHN_post_ − VHN_lesion_. Furthermore, the percentage surface microhardness recovery (% SMHR) [11, 12] and change (% SMHC) [13] were calculated as follows (IL: indentation length; mean diagonal length of both diagonals): % SMHR = (IL_lesion_ − IL_post_)/(IL_lesion_ − IL_sound_) × 100%; % SMHC = (VHN_post_ − VHN_lesion_)/VHN_lesion_ × 100%. Positive ΔVHN, % SMHR, and % SMHC values indicated lesion rehardening, while negative values were indicative of further demineralization. The analyses of % SMHR and % SMHC were conducted as part of a post hoc analysis to determine if variables derived from the raw data can impact conclusions.

### 2.8. Statistical Analysis

The data were tested for normal distribution (Shapiro-Wilk test). The variables VHN_sound_, VHN_lesion_, ΔVHN, % SMHR, and % SMHC were calculated for each specimen and analyzed using a two-way ANOVA with factors for “lesion severity” and “fluoride concentration” and their interaction. ΔVHN was considered the primary variable. Where significant differences were indicated, the individual means were analyzed by Fisher's least significant difference test. The significance level for the analyses was set at 5%.

## 3. Results

The results and statistical analyses for the sound enamel and lesion baseline VHN data can be found in Table 2. There were no differences in VHN_sound_ between treatment groups (*p* = 0.988). Increasing demineralization times led to decreased VHN_lesion_ values with the four chosen lesion severities exhibiting different degrees of surface softening (*p* < 0.001).

**Figure 1:**
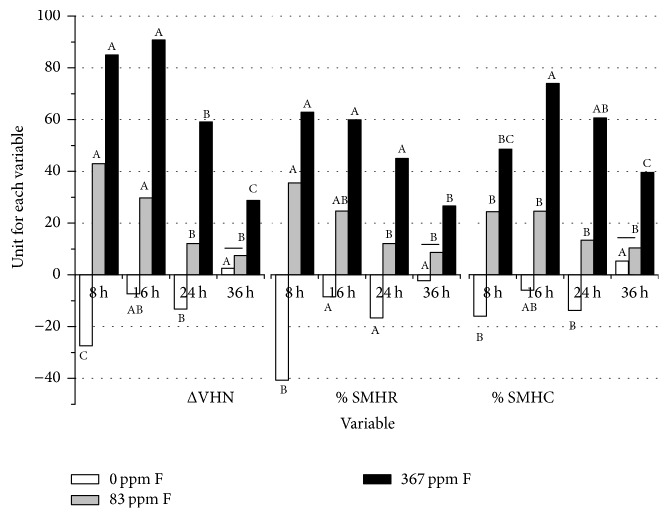
Least square means for all post-pH cycling variables by lesion severity and fluoride concentration. Error bars were omitted for better clarity. Horizontal lines indicate fluoride concentration comparisons within each lesion severity which were not statistically significantly different. Different capital letters highlight statistically significant differences within the same fluoride concentration between lesion severities.

Figure 1 presents the data and results of the statistical analyses for all post-pH cycling variables by lesion severity and fluoride concentration. The lesion severity × fluoride concentration interaction was significant for ΔVHN, % SMHR, and % SMHC (all *p* < 0.001). All variables were able to discern (all *p* ≤ 0.04) or not (only 36 h lesion: 0 versus 83 ppm fluoride, all *p* ≥ 0.20; horizontal lines) between the tested fluoride concentrations in a dose-response manner within each of the four lesion severities.

Baseline lesion severity affected the response differential, that is, the numerical difference of 0 versus 83 versus 367 ppm fluoride, which also depended on the variable. For ΔVHN, differences of 0 versus 367 ppm fluoride became progressively smaller with increasing lesion severity [ΔVHN (8 h) = 112; ΔVHN (16 h) = 98; ΔVHN (24 h) = 72; ΔVHN (36 h) = 26]. A similar trend was observed for % SMHR (104 versus 68 versus 62 versus 29), although not for % SMHC (65 versus 80 versus 75 versus 34). There was more agreement between variables when comparing 0 versus 83 ppm as the numerical differences decreased with increasing lesion severity for all variables. When comparing 83 versus 367 ppm, no clear lesion severity effect was noted for any variable, with the largest numerical difference observed for all variables for the 16 h lesion, followed by the 24 h lesion. For both ΔVHN and % SMHR, the 8 h lesion yielded larger differences than the 36 h lesion, which was reversed for % SMHC (data are not shown but can be derived from Figure 1).

Overall, ΔVHN was most able to discern between lesion severities (Figure 1; capital letters), although virtually identical trends were observed for % SMHR. % SMHC was different in that no differences were observed between lesion severities for 83 ppm fluoride, although trends were similar. Likewise, the rank order for 367 ppm fluoride was different compared to ΔVHN and % SMHR. Only the rank order for 0 ppm fluoride was comparable between all variables.

## 4. Discussion

The present laboratory study was primarily concerned with investigating the effect of enamel caries lesion baseline severity on fluoride dose-response of very early caries lesions under pH cycling conditions. The present pH cycling model was chosen to allow for further surface softening as well as rehardening to be observed and its relative short duration (5 d) to prevent complete rehardening of lesions while also being able to discriminate between treatments. The chosen lesions were very shallow, early caries lesions (lesion depth ≤ 50 *μ*m when extrapolating prior data [14]) which under clinical conditions would not likely be detected or considered questionable. Consequently, Vickers surface microhardness was chosen ahead of the “gold standard” technique, transverse microradiography (TMR), due to Vickers' greater discernibility in shallower lesions. However, it must be borne in mind that although very good agreement was shown between surface microhardness techniques and TMR in several studies and for a range of lesion types and severities [12–17], hardness techniques do not measure mineral content per se and no attempt was made presently to correlate hardness changes with mineral gain or loss.

The present findings have provided further evidence to the varying efficacy of fluoride in caries prevention. Shallower lesions were found to be considerably more responsive to fluoride than deeper lesions (ΔVHN and  % SMHR data), despite the former's greater susceptibility to further demineralization (Figure 1, 0 ppm F data) [8], the latter's greater capacity for remineralization [5–8], and the greater ability of the latter to absorb more fluoride (based on the virtue of being more demineralized [18]). These aspects would have predicted opposite results than those observed presently. However, the aforementioned limitations of these studies (e.g., no fluoride dose-response) make it difficult to compare between studies. Likewise, although similar in design, the sole, comparable pH cycling study [9] considered more advanced lesions, although only in a laboratory research sense. In this prior study, it is likely that the lesion surface layer had a marked effect on fluoride dose-response. Acting as a diffusion barrier it controls the in- and outflow of minerals; however, incorporation of fluoride can lower porosity to the point of lesion arrest due to hypermineralization. Presently, lesions were predominantly surface-softened (based on extrapolation of data from a similar study incorporating TMR [14]) which may explain their greater capacity for re- and further demineralization. The findings for 0 ppm fluoride are in agreement with a previous mechanistic study [8] which has shown that initially less demineralized lesions are more susceptible to further demineralization than initially more demineralized ones. One explanation may lie in the fact that more demineralized lesions have lost comparatively more soluble minerals (i.e., those containing sodium, magnesium, and carbonate) during demineralization, leaving behind more acid-resistant minerals (i.e., those containing fluoride) than early, less demineralized lesions. Therefore, the inherent solubility of enamel changes during demineralization and proportionally decreases with increasing demineralization time. Interestingly, the findings for fluoride are not in agreement with previous observations [9], as initially shallower lesions rehardened more than initially deeper ones. It can only be speculated that the (complete) absence of a well-defined surface layer in the 8 and 16 h lesions promoted greater mineral influx, thereby allowing for more rehardening to occur in the presence of fluoride. The more demineralized lesions were comparatively less responsive and likely due to their more developed lesion structure (surface zone, lesion body), which was also demonstrated in situ [6]. As mentioned before, TMR was not employed presently due to its lack of sensitivity in shallower lesions, although this is undoubtedly one of the limitations of the present study as no data on surface zone mineral density were derived. Surface microhardness measurements provide information about structural integrity and are complementary to TMR but not a like-for-like replacement.

The collection of hardness data from a range of lesion severities also presented an opportunity to consider multiple variables and to compare their ability to discern between fluoride concentrations and to investigate the effect of baseline lesion severity. For Vickers surface microhardness (Vickers-SMH), typically only ΔVHN is being considered, although both % SMHR and % SMHC have been used in the interpretation of data [12, 13]. % SMHR was initially proposed for Knoop-SMH and takes into account sound, demineralized, and posttreatment indentation lengths [11]; however, the present study has shown that it correlates well with ΔVHN, which is based on hardness number changes of posttreatment minus demineralized specimens. % SMHC is somewhat comparable to both as it considers percentage changes in hardness numbers of posttreatment versus demineralized specimens. Overall, considering each lesion severity separately, no differences were observed in their ability (or lack thereof) to discern between fluoride concentrations. Therefore, all variables are justifiable. However, when comparing within fluoride concentrations between lesion severities, data interpretation becomes more complex as different conclusions can be drawn depending on the variable (Figure 1). ΔVHN was able to differentiate the most between lesions, whereas % SMHR, although showing similar patterns to ΔVHN, showed the least ability to highlight differences. Using % SMHC, however, would allow different conclusions to be drawn. This highlights the need for a consensus among researchers as often variables (or entirely new measures) are being introduced to highlight potential findings that would not exist if it was not for the new variable or measure.

The present study undoubtedly has limitations, with being a laboratory investigation representing perhaps the most important shortcoming. The present inability to monitor pro- and regression of in vivo caries lesions as studied presently, however, leaves researchers little choice but to withdraw from the clinical scenario and focus on artificial caries instead.

## 5. Conclusions

Bearing in mind the laboratory nature of the present study, it can be concluded that lesion baseline severity impacts the extent of the fluoride dose-response. Fluoride is most effective when introduced as early as possible in the caries process. Furthermore, care must be taken when interpreting data as different variables can lead to different conclusions.

## Figures and Tables

**Table 1:** Daily pH cycling regimen.

Duration	Specimen treatment
1 min	Intervention
60 min	Remineralization
60 min	Demineralization
60 min	Remineralization
1 min	Intervention
60 min	Remineralization
60 min	Demineralization
60 min	Remineralization
1 min	Intervention
(Overnight)	Remineralization

**Table 2:** Least square means and results of the statistical analyses for VHN_sound_ and VHN_lesion_.

Lesion severity	Fluoride concentration	VHN_sound_	VHN_lesion_	
8 h	0 ppm	347	176	A^1^
	83 ppm	347	178	
	367 ppm	346	180	

16 h	0 ppm	344	122	B
	83 ppm	346	123	
	367 ppm	344	124	

24 h	0 ppm	344	93	C
	83 ppm	349	95	
	367 ppm	343	96	

36 h	0 ppm	343	72	D
	83 ppm	345	73	
	367 ppm	346	74	

SEM^2^		4	3	

Lesion severity^3^		*0.951*	*<0.001*	
Fluoride concentration		*0.699*	*0.578*	
Lesion severity × fluoride concentration		*0.988*	*1.000*	

^1^Statistically significant differences between lesion severity groups are highlighted by different capital letters. The chosen four demineralization times led to four distinct extents of surface softening.

^2^Standard error of the least square mean (identical for each variable as two-way ANOVA will yield a pooled SEM); value presented only once per variable for better clarity.

^3^ *p* values for each factor and interaction between factors.
